# Elucidating Key Motifs Required for Arp2/3-Dependent and Independent Actin Nucleation by Las17/WASP

**DOI:** 10.1371/journal.pone.0163177

**Published:** 2016-09-16

**Authors:** Ellen G. Allwood, Joe J. Tyler, Agnieszka N. Urbanek, Iwona I. Smaczynska-de Rooij, Kathryn R. Ayscough

**Affiliations:** Department of Biomedical Science, Firth Court, University of Sheffield, Sheffield S10 2TN, United Kingdom; Institute of Biology Valrose, FRANCE

## Abstract

Actin nucleation is the key rate limiting step in the process of actin polymerization, and tight regulation of this process is critical to ensure actin filaments form only at specific times and at defined regions of the cell. Arp2/3 is a well-characterised protein complex that can promote nucleation of new filaments, though its activity requires additional nucleation promotion factors (NPFs). The best recognized of these factors are the WASP family of proteins that contain binding motifs for both monomeric actin and for Arp2/3. Previously we demonstrated that the yeast WASP homologue, Las17, in addition to activating Arp2/3 can also nucleate actin filaments de novo, independently of Arp2/3. This activity is dependent on its polyproline rich region. Through biochemical and in vivo analysis we have now identified key motifs within the polyproline region that are required for nucleation and elongation of actin filaments, and have addressed the role of the WH2 domain in the context of actin nucleation without Arp2/3. We have also demonstrated that full length Las17 is able to bind liposomes giving rise to the possibility of direct linkage of nascent actin filaments to specific membrane sites to which Las17 has been recruited. Overall, we propose that Las17 functions as the key initiator of de novo actin filament formation at endocytic sites by nucleating, elongating and tethering nascent filaments which then serve as a platform for Arp2/3 recruitment and function.

## Introduction

Las17 is the primary activator of Arp2/3-driven actin nucleation in the budding yeast *Saccharomyces cerevisiae*, and is required for membrane invagination during endocytosis [1–3]. Its mammalian orthologues, the WASP family of proteins, which include WASP, N-WASP, WAVE, and WASH have also been studied extensively as activators of Arp2/3-driven actin polymerization [4–7]. Las17 has a similar domain structure to mammalian WASP having an N-terminal WH1 domain, a central proline rich region and a C-terminal WCA region. In both Las17 and WASP, actin nucleation activity is attributed to this C-terminal region that contains a WH2 domain that can bind monomeric G-actin and a ‘central+acidic’ region that has been shown to interact with Arp2/3 [4, 7]. Las17 does not however contain a GTPase binding region so is not considered to be regulated by binding of rho-family GTPase proteins.

The central region of both Las17 and WASP is rich in proline residues and has been shown to bind a number of SH3 domain-containing proteins. In an earlier study we demonstrated that, in addition to SH3 domain binding, Las17 polyproline (PP) region not only binds directly to actin, but it is also able to nucleate actin filaments in the absence of its own WCA domain and of Arp2/3 [8]. Such a mechanism would allow generation of ‘mother’ filaments at key cell sites, which could then recruit Arp2/3 to drive further rounds of nucleation and polymerization. Deletion of a 36 amino acid stretch at the C-term end of the polyproline region led to a marked reduction in actin binding in a yeast two-hybrid assay used to measure the interaction [8]. This 36 amino acid region contained two tracts of 5 contiguous proline residues and led to us to investigate the importance of prolines for direct actin binding. A 28mer peptide encompassing both of these proline tracts was able to increase the elongation rate of actin filaments but did not nucleate in the absence of the rest of the polyproline region. Mutation of two prolines to alanine in each of the tracts did however, render the peptide inactive towards actin. This led us to suggest that multiple proline tracts in the region contributed low levels of actin binding, which together were able to facilitate nucleation and elongation of actin filaments [8]. Importantly, this polyproline-mediated actin regulatory function of Las17 was critical in vivo. Cells expressing Las17 with mutations in just 2 prolines (*las17 PP506*,*507AA*), were temperature sensitive with defects early in the endocytic process at a stage prior to invagination. We therefore hypothesized that Las17-mediated actin nucleation was able to generate ‘mother’ filaments that could then recruit Arp2/3. When recruited to these existing filaments and bound to its nucleation promoting factor Las17, Arp2/3 could then drive a rapid burst of actin nucleation to facilitate membrane invagination required for endocytosis.

While our previous study demonstrated a role for the polyproline region in generating ‘mother’ filaments for Arp2/3 recruitment, questions remained as to the motifs within this region that confer the actin nucleating and elongating activity and also, how the function of the polyproline region interfaces with that of the better studied WCA region.

## Results

### Assessing the contributions of PP and WCA regions for Arp2/3 independent and dependent Las17 actin nucleation

**Fig 1:**
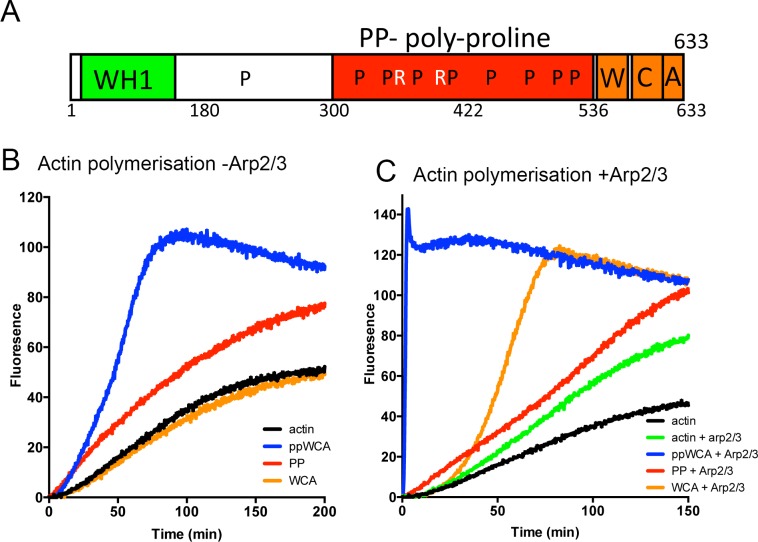
**Interplay between Las17 polyproline and WCA domains** (A) schematic showing domain structure of Las17. WH1 –WASP homology-1 region; W–WH2 or WASP homology 2 region; C–central region; A–acidic region; P denotes tracts of 5 proline residues. R denotes those tracts with paired arginines in close proximity. Actin polymerization assays following increased fluorescence due to incorporation of pyrene-labeled actin into filaments were carried out to assess the effect of Las17 fragments in the absence (B) and presence (C) of Arp2/3. Shown are representative experiments. Each assay has been repeated independently a minimum of three times.

In order to investigate the role of the polyproline region in the context of the G-actin binding WH2 domain and the C-terminal acidic domain, relevant constructs were generated, expressed recombinantly and purified as described. Fig 1A highlights different domains of the protein and the regions included in constructs.

Pyrene-actin filament incorporation assays were followed fluorimetrically to determine whether the addition of WCA fused to the PP region, or simply co-incubation of PP and WCA separately, in assays affected the nucleation and elongation of actin filaments. As shown (Fig 1B), actin alone in the presence of polymerization salts shows a lag period, followed by filament elongation, finally reaching a steady state. In the presence of the PP region alone (red line), the lag period is reduced indicating PP is nucleating filaments as had been previously observed. When PP-WCA was expressed, the presence of WCA did not reduce the lag period and therefore did not contribute to actin nucleation, but it did enhance actin elongation at lower monomeric actin concentrations. The addition of the WCA fragment alone (0.3 μM WCA) to actin (3 μM) slightly reduced the polymerization rate supporting the large amount of data demonstrating the region to be G-actin binding [9]. Thus, when not fused to the polyproline domain, WCA does not contribute positively to polymerization in the absence of Arp2/3.

The importance of PP and WCA were then analysed in the presence of Arp2/3 to investigate whether the Arp2/3-dependent and independent mechanisms have fundamentally different requirements for the domains. As shown in Fig 1C, in the presence of Arp2/3, actin alone shows a slight increase in polymerization rate. The addition of the PP only fragment allows nucleation but this does not appear to be any greater than in the absence of Arp2/3. The WCA domain alone does not activate Arp2/3-nucleating function, though it does enhance elongation. However the combination of the polyproline and the WCA domain in a single fragment induces a very dramatic increase in actin nucleation and elongation rates confirming previous data that in order to function as a nucleator Arp2/3 requires motifs in both PP and WCA domains [10].

### The minimal sequence requirement of the polyproline region for Las17 nucleation

Our previous work had suggested contributions for actin binding in both N- and C-terminal halves of the Las17 PP domain. We therefore generated truncations of the PP-WCA domain to refine the region absolutely required for actin nucleation activity rather than just increasing the elongation rate. We also included a construct (180–633) incorporating the single proline tract that lies upstream of the main polyproline region to determine whether this tract adds further activity to nucleation or elongation.

**Fig 2:**
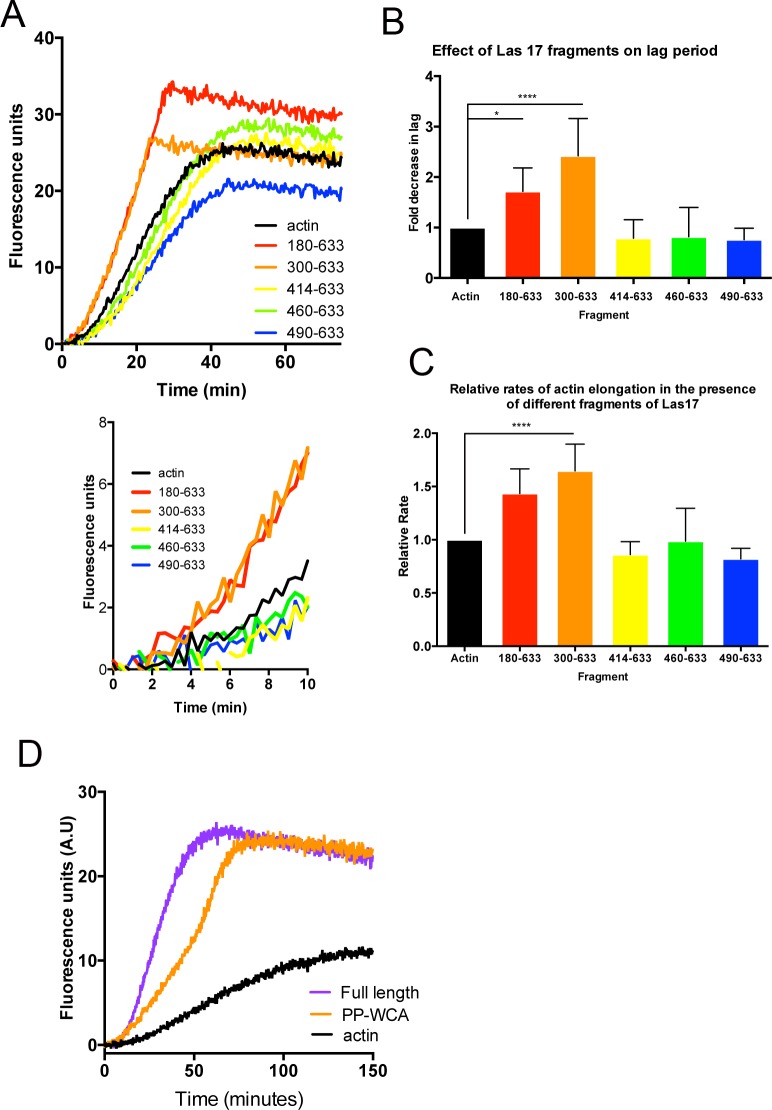
Analysis of truncated fragments of Las17 to determine regions required for nucleation in the absence of Arp2/3. (A) Five fragments of Las17 were generated containing 9 (180–633), 8 (300–633), 4 (414–633), 3 (460–633) or 2 (490–633) tracts of 5 prolines, fused to the WCA region. These were used in a pyrene-actin based polymerization assay to determine the parts of the polyproline region required for actin filament nucleation and elongation. The lower graph shows the first 10 minutes of polymerization to highlight the effect of the fragments at early time points more clearly. The effect of the fragments on (B) the reduction in the lag phase relative to actin alone and (C) the relative rates of F-actin elongation were analysed from at least three independent experiments with each fragment. In statistical analysis the Las17 180–633 and 300–633 fragments show significant reductions in lag phase (p value <0.0001) and elongation (p value <0.0001) using one-way ANOVA when compared to actin alone. ** highlights significance based on Dunnetts multiple comparison test in one-way ANOVA. (D) Pyrene-actin based polymerization assay were used to compare the activity of the PP-WCA fragment (residues 300–633) with full length Las17 purified from yeast in the absence of Arp2/3.

As shown in Fig 2A, only the PP-WCA (300–633) and the N-terminally extended 180–633 contained actin-nucleating activity. All other shorter constructs that contained either 2, 3 or 4 tracts of 5 prolines (5xPP) appended to the WCA domain, but lacking residues 300–413, did not affect either actin nucleation or filament elongation relative to actin. Fig 2A shows a representative experiment. Combination of data from multiple experiments (n≥3) allowed a clearer determination of the effect of the different fragments on both nucleation and elongation (Fig 2B and 2C). These data showed that the effect of the 300–633 fragment was the most robust over all experiment repeats. It also indicated that the inclusion of the most N-terminal proline tract does not appear to add any further activity to the 300–633 fragment, at least in the context of this assay. The combined data also supported the idea that the smaller fragments, lacking the N-terminal polyproline region, are not able to enhance nucleation or elongation.

In a previous study [10], it was demonstrated that full length Las17 potently activated Arp2/3-dependent actin nucleation compared to the WCA domain alone. To determine whether full length Las17 purified from yeast shows different behaviour from the PP-WCA fragment in the absence of Arp2/3, a pyrene-actin incorporation assay was performed. As shown (Fig 2D), the full length protein shows no increase in nucleation capacity compared to the shorter fragment but there is an enhanced filament polymerization rate.

### The importance of paired basic residues in actin nucleation with the polyproline region

In 2015 a second G-actin binding site was identified in Las17 and was shown to contribute to Arp2/3-dependent actin polymerization [11]. This binding site was suggested to comprise paired arginine residues at amino acids 349, 350 and at 382, 383. When these residues were mutagenised the PP-WCA region was reduced in its ability to nucleate and elongate actin filaments in the presence of Arp2/3. Given that these residues lie in the N terminal (300–414) part of the PP domain we had identified above as relevant for Las17 actin nucleation function we aimed to confirm the role of the paired arginines in G-actin binding and to address their importance in Arp2/3-independent actin nucleation.

**Fig 3:**
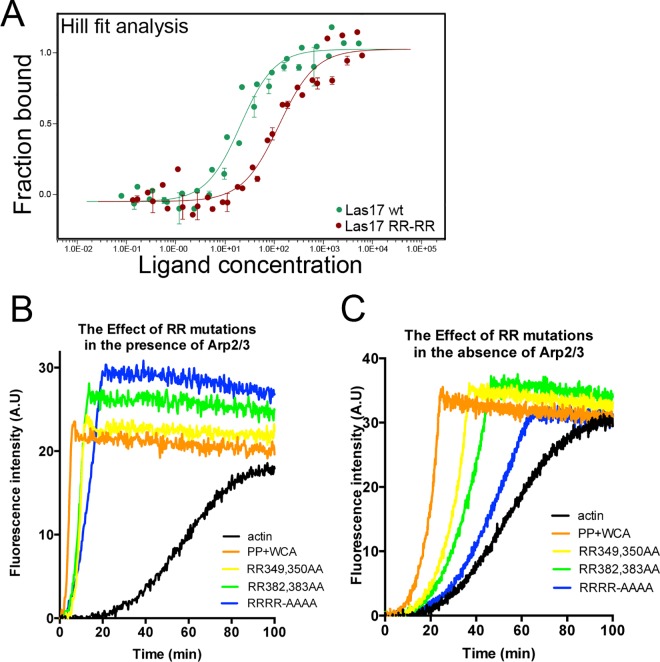
The importance of paired arginine residues for actin binding and polymerization. (A) Microscale thermophoresis was used to measure the binding affinity of a wild type or RRRR-AAAA mutant fragment of Las17(300–422) for G-actin (50 nM). The effect of both single and double arginine pair mutations on actin polymerization was assessed in the context of the longer PP-WCA fragment in the presence (B) or absence (C) of Arp2/3. Independent assays performed ≥3 times.

Microscale thermophoresis was used, as described in Experimental procedures, to compare G-actin binding affinity of the wild type 300–422 fragment of Las17 to that carrying mutations to alanine at both RR pairs (i.e RR349,350AA and RR382,383AA). Mutation to alanine was selected to reduce the possibility of other major structural changes in the region. As shown in Fig 3A, the double arginine pair mutation causes a marked reduction in binding affinity compared to the wild type fragment (Kd wt 24.9 nM ±3.6, Hill coefficient 1.25; Kd RRRR mutant 148.3 nM ±24.9, Hill Coeffieicent 1.18). This confirms the importance of the basic residues in this fragment for G-actin binding. It is important however to note that detectable binding is still present in the double RR pair mutant suggesting the presence of other weaker actin-binding capacity in the region.

Having confirmed the importance of the paired arginine residues for G-actin binding we then tested the relevance of these in a pyrene-based actin polymerization assay with mutations being generated in the PP-WCA fragment. In the presence of Arp2/3 we confirmed the previous work and demonstrated that there was a clear reduction in nucleation and elongation rates of actin when actin was incubated with the mutant proteins (Fig 3B) [11]. The single paired RR mutations gave an intermediate phenotype in this assay.

We then addressed the importance of the RR residues in the absence of Arp2/3. As shown in Fig 3C, the RRRR-AAAA mutation had a very dramatic effect on both nucleation and elongation rate. Again the single pair mutants had an intermediate effect. The data suggests that the RR pairs are important for nucleation, and this effect appears particularly critical in the absence of Arp2/3.

### What is the effect of mutagenising the RR residues pairs in cells?

**Fig 4:**
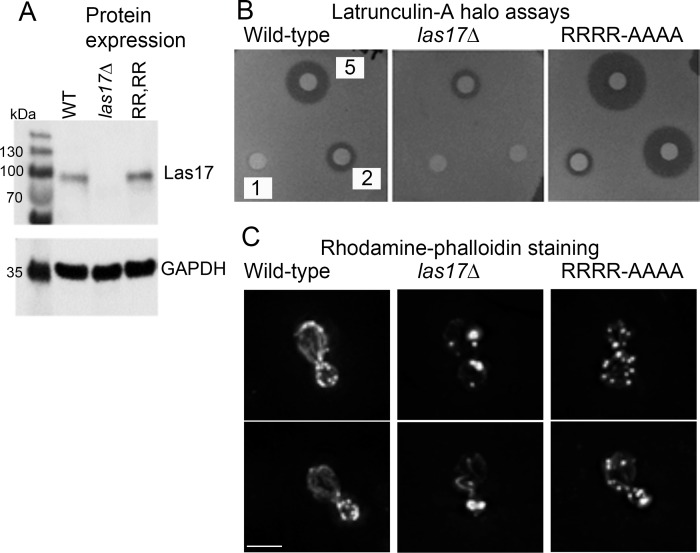
The effect of Las17 RRRR-AAAA expression in cells. (A) A mutant form of *las17* encoding Las17 RR349,350 AA;RR383,383 AA was integrated into the genome. Expression levels were tested using anti-Las17 antibodies. Numbers indicate size of standard markers. (B) Halo assays were used to determine latrunculin-A sensitivity of the mutant strain compared to wild-type and *las17* deletion strains. Numbers indicate the concentration of latrunculin-A (mM) applied to the filter discs. (C) Rhodamine-phalloidin staining was used to determine organization of F-actin in cells. Two cells are shown for each strain. Bar = 5 μm.

While the paired basic residues appear critical for Las17 mediated actin nucleation in vitro, it is then relevant to address the importance of these residues in vivo. In order to test this role, an RRRR-AAAA mutation was integrated into the genome. Expression levels of wild type and mutant protein were similar indicating that the mutations do not affect stability of the protein (Fig 4A).

Given that the RR residues are important for G-actin binding in vitro, their mutation would also be expected to lead to a reduced capacity of Las17 to bind monomeric actin in cells and so potentially an increase in G-actin levels. Increased G-actin levels have previously been correlated to an increased sensitivity to the actin binding compound latrunculin-A [12]. This idea was tested using different concentrations of latrunculin-A spotted onto filters on a nascent lawn of yeast cells. Inhibition of growth generates halos around the filter discs and the size of the halo relates to drug sensitivity. As shown in Fig 4B deletion of *las17* causes resistance of cells to latrunculin-A compared to wild-type cells indicating increased actin stability. In contrast, the RRRR-AAAA mutation that binds G-actin less well in vitro causes an increased sensitivity to latrunculin-A supporting the idea that this motif is also involved in G-actin binding in vivo.

Given the effect of the RR mutations on actin polymerization in vitro, the effect on actin organization in vivo was then investigated. As previously reported, deletion of *las17* results in a major disruption of actin [2]. Rather than many small polarized cortical actin patches, there are larger actin clumps that are less well polarized (Fig 4C). This stabilized, actin clump phenotype also fits with the enhanced resistance to latrunculin-A. The actin in cells expressing the mutant was markedly different from the deletion strain. Patches were similar in size to those in the wild-type strain. In terms of patch organization, in the wild-type cells, patches in budded cells are largely polarized (96%). In the mutant, there was a shift towards depolarization with 81% cells showing all, or partial, patch depolarization (Fig 4C).

### Mutagenesis of the RR pairs affects endocytosis prior to Arp2/3 recruitment

If the role of the RR pairs is associated specifically with Arp2/3 function, then early stages of endocytosis should proceed normally and defects would be expected to be concomitant with Arp2/3 recruitment and membrane invagination. If however these residues function in Las17-mediated actin nucleation, independent of and prior to Arp2/3 then an earlier endocytic defect would be predicted.

**Fig 5:**
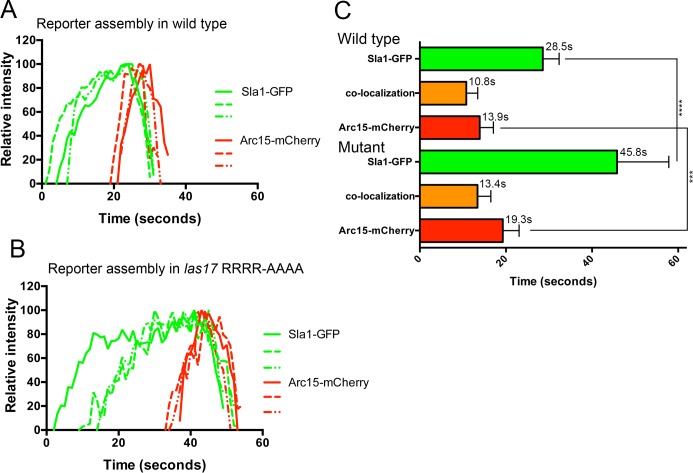
The effect of the Las17 RRRR-AAAA mutation on recruitment of key endocytic proteins. Strains expressing reporters for early stages of endocytosis (Sla1-GFP) and for Arp2/3 (Arc15-mCherry) were generated. The behaviour of reporters was analysed in otherwise wild-type cells (A) or in cells harbouring an integrated mutant allele of Las17 (*las17 RRRR-AAAA*) (B). The profiles show fluorescence intensity over time for 3 different patches analysed. A total of 30 patches were analysed for each strain and the combined data are shown in (C). Error bars denote standard deviation. Sla1-GFP has a significantly different lifetime between the strains (**** students t-test p<0.0001). Arc15-mCherry has a significantly different lifetime between the strains (*** students t-test p = 0.0009). Mean lifetimes are given. n = 30.

To determine the stage of endocytosis requiring the basic residues for actin nucleation, the relative behaviours of two endocytic markers was investigated. Sla1-GFP was used as a reporter of early endocytosis and Arc15-mCherry was used to report directly on recruitment and disassembly times of the Arp2/3 complex from endocytic sites. As shown in Fig 5A, in wild-type cells Sla1-GFP is recruited to endocytic sites 15–20 seconds prior to arrival of Arc15-mCherry. The recruitment of Arc15 is very rapid with maximal intensity being reached only a few seconds after the signal is first detected. Disassembly of Sla1-GFP from sites just precedes that of Arc15. Three independent examples of intensity profiles are depicted here, but combined lifetime data for 30 Sla1-GFP, Arc15-mCherry patches and their co-localization times is shown in Fig 5C. In the presence of the Las17 RR349,350AA, RR382,383AA mutant, there is a significant change in lifetime of Sla1-GFP at the endocytic sites with an increase in the mean lifetime from 28.5 seconds to 45.8 seconds (students t test p value <0.0001) and also a greater variability in these lifetimes. The time between Sla1-GFP and Arc15-mRFP recruitment is also significantly greater. The lifetime of Arc15-mCherry was also increased (13.9–19.3 seconds; students t test p value <0.0001) indicating that effects of the mutations also contribute to defects following recruitment of Arp2/3. These data are comparable to those obtained by Feliciano and colleagues when studying the effect of the paired arginine residues mutations on Sla1-RFP and Abp1-RFP reporters when each was compared to Las17-GFP [11].

The data clearly shows that the RRRR-AAAA mutation has a marked effect prior to Arp2/3 recruitment. Overall the in vivo data provides strong evidence for the importance of the RR-defined actin binding sites within the polyproline region of Las17 for functioning at early stages of endocytosis.

### The role of polyproline tracts within the PP region

The in vitro data from our work and from others, strongly supports the role of the paired basic residues in G-actin binding and in actin nucleation. However, the yeast two-hybrid and other in vitro assays previously reported by our lab, also demonstrated a clear interaction between the Las17 polyproline region and actin that was highly dependent on proline residues [8]. It was also clear that mutation of two prolines (PP506,507AA) caused a severe actin and endocytic phenotype in vivo demonstrating the importance of the proline tracts within a cellular context. A significant question then emerges as to the functional role of the proline tracts themselves.

**Fig 6:**
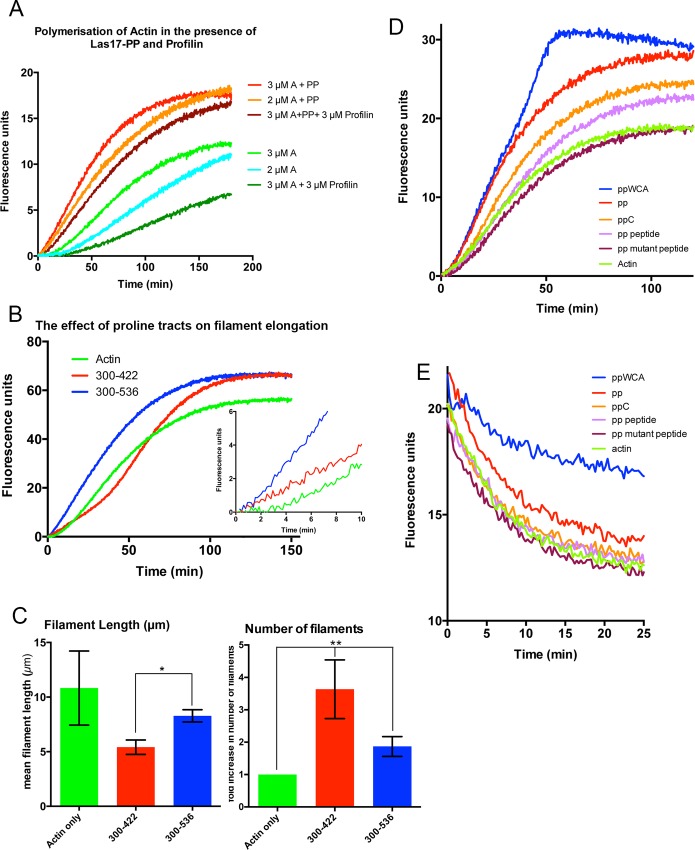
The role of proline tracts in the polyproline region. (A) Pyrene assays were used to assess the possible contribution of profilin to the Las17 PP-mediated actin nucleation. Profilin was added to the assays in the presence or absence of Las17 PP and actin–A. (B) The effect of proline tract number on filament elongation was assessed by incubating actin in the presence of Las17 fragments carrying 4 (300–414) or 8 (300–536) proline tracts. Inset shows early time points to highlight effects on nucleation. (C) The length of actin filaments formed after 30 minutes polymerization in the presence of the 4- or 8-proline tract fragments was assessed by visualizing filaments using fluorescence microscopy. Multiple fields of view were recorded and the length (C-left) and number (C-right) of filaments recorded. The difference between filament lengths formed in the presence of the short or long fragment was significant (*), p value in Mann Whitney test 0.029, from 4 independent experiments. Number of filament lengths measured for each condition in each experiment ≥102. The filament numbers per unit image area were counted and normalized to actin. The graph shows fold increase in filament number for the two Las17 fragments tested. The difference in filament number is statistically significant (P = 0.0036) in a 1 way ANOVA analysis (n = 3 independent experiments). The effect of increasing proline tract number on elongation rate (D) and depolymerization rate (E) was recorded as described for fragments carrying increasing numbers of polyproline tracts. These experiments used actin at 5 μM. Fragments used: PP peptide (residues 501–528); mutant peptide (residues 501–508 with alanine for proline substitutions at residues 506,507, 525,526); ppC–which carries the C-terminal 4 proline tracts (residues 414–536); PP (full proline tract 300–536) and PP-WCA (full proline tract and the WCA region).

The presence of tracts of proline residues within Las17 has led to the suggestion that this region may interact with profilin and that this binding could somehow drive actin nucleation or elongation. However, at least in the case of Las17, a number of distinct lines of evidence indicate that a direct profilin-Las17 interaction is not occuring. First, none of the proline tracts in Las17 is longer than 5 consecutive proline residues. The previously measured binding affinity of profilin for a Pro6 peptide or a 5Pro-Gly-5Pro peptide were in the mM range which was 10–100 fold less than for a proline decamer and therefore interactions would be unlikely at physiological concentrations of either protein [13]. Second, several yeast proteomic studies focusing on identification of protein-protein interactions, have not reported interactions between Las17 and profilin [10, 14]. Third, use of the yeast two-hybrid assay demonstrated a Las17 PP-actin binding interface between subdomains 3 and 4 on actin, while profilin itself has been demonstrated to bind between actin subdomains 1 and 3 indicating that profilin is not bridging the interaction between Las17-PP and actin [8, 15]. However, to confirm that profilin does not impact on nucleation mediated by the Las17 proline-rich region, polymerization of actin was followed in the presence or absence of the Las17 polyproline fragment and profilin. As shown in Fig 6A, the addition of the PP region alone (without WCA) leads to nucleation and enhanced elongation of F-actin (red line compared to green line). The addition of profilin to these assays caused a reduction in nucleation and elongation consistent with sequestration of actin. Intriguingly, the effect of profilin when actin is polymerized in the presence of Las17-PP appears diminished when compared to actin polymerized just in the presence of salt also suggesting there is reduced availability of monmer for profilin binding. The reduction in filament polymerization is similar to that observed when a reduced concentration of actin (2 μM) is available for polymerization. Our data demonstrate that the presence of profilin does not contribute to the nucleation function of Las17 in the absence of Arp2/3.

Given that profilin does not contribute to the Las17 actin polymerization mechanism we next considered intrinsic properties of the region itself and whether the role of the C-terminal polyproline tracts (C-PP) might be tightly coupled to the nucleation role of the basic residues in the N-terminal portion of the region. We hypothesized that, through weak and co-operative associations with F-actin, the C-terminal proline tracts might serve to stabilize or tether a growing filament, and in doing so could promote its elongation. We addressed two key questions. (1) What is the impact on actin nucleation of having a greater number of proline tracts and (2) how does proline tract number influence elongation and depolymerisation of actin filaments.

To address the first question we compared actin nucleation in the presence of the N-terminal PP peptide, Las17(300–422) and the longer PP fragment (300–536). The former fragment contains 4 tracts of 5 prolines while the second construct contains eight tracts of 5 prolines. As shown in Fig 6B inset, at very early time points both fragments appear to nucleate. However, while the longer fragment shows rapid filament elongation, the shorter N term-PP fragment does not effectively elongate filaments and rather seems to sequester these nuclei or short filaments. This result would then predict that, at an early time point (30 minutes), filaments generated in the presence of the N-term PP fragment would be shorter than those generated in the presence of the longer PP fragment. To test this idea, actin was polymerized in the presence of Alexa488nm actin as described in the Methods. At 30 minutes, samples were taken and filaments imaged microscopically. Actin filament lengths were measured, and also the number of filaments forming per unit area were counted. As shown in Fig 6C, in the absence of Las17 fragments, filaments that do form tend to be longer (left graph), but far less numerous (right graph), presumably because nucleation is rare while elongation of filaments is favourable. In the presence of the N-term PP fragment the length of filaments was significantly shorter than those generated in the presence of the longer PP fragment (mean filament length Las17(300–422) 5.53 μm; Las17(300–536) 8.54 μm). There were also more filaments present in the samples containing the N-term PP fragment. Together these data support the idea that the C terminal proline tracts might function to bind and stabilize growing filaments to allow their more effective elongation.

If the proline tracts do interact with growing filaments, then this would also predict that a greater number of tracts should lead to both increased elongation and potentially stabilization of filaments. To test this a number of fragments of Las17 were incubated with actin in pyrene-based assays. These fragments had increasing numbers of proline tracts and included: PP peptide (a 28 mer peptide carrying the C-terminal 2 proline tracts); PP mutant peptide (the same 28mer peptide as above but with the two prolines in each tract mutagenized to alanines); PP-C–a fragment with the 4 most C-terminal proline tracts; PP–with 8 proline tracts; PP-WCA with 8 proline tracts and the WCA domain. As shown in Fig 6D the rate of elongation increases with increasing proline tract number and over all a higher level of polymerization is reached. Following dilution of samples to assess depolymerization, the majority of filaments disssembled with similar kinetics to those generated only in the presence of salt (Fig 6E). However the presence of the full PP fragment (300–536) and the longer PP-WCA fragment both appeared to stabilize filaments. This effect was particularly marked for the PP-WCA fragment and indicates the possibility that the presence of the WH2 domain might play a significant role in the rate of filament turnover.

### Las17 tethering to membranes

A critical question concerning spatiotemporal regulation of actin nucleation is how both the nucleator itself, and the filaments once generated, are held in close proximity to the membrane where they are to function. Las17 localizes to endocytic sites before the majority of its known interaction partners and the factors that facilitate its localization are not well understood. For example, deletion of the genes encoding various SH3 domain proteins with which Las17 is known to interact including Sla1 and Bbc1, do not result in Las17 mislocalization from the plasma membrane [1]. Las17 plasma membrane localization is also not inhibited by disruption of actin.

**Fig 7:**
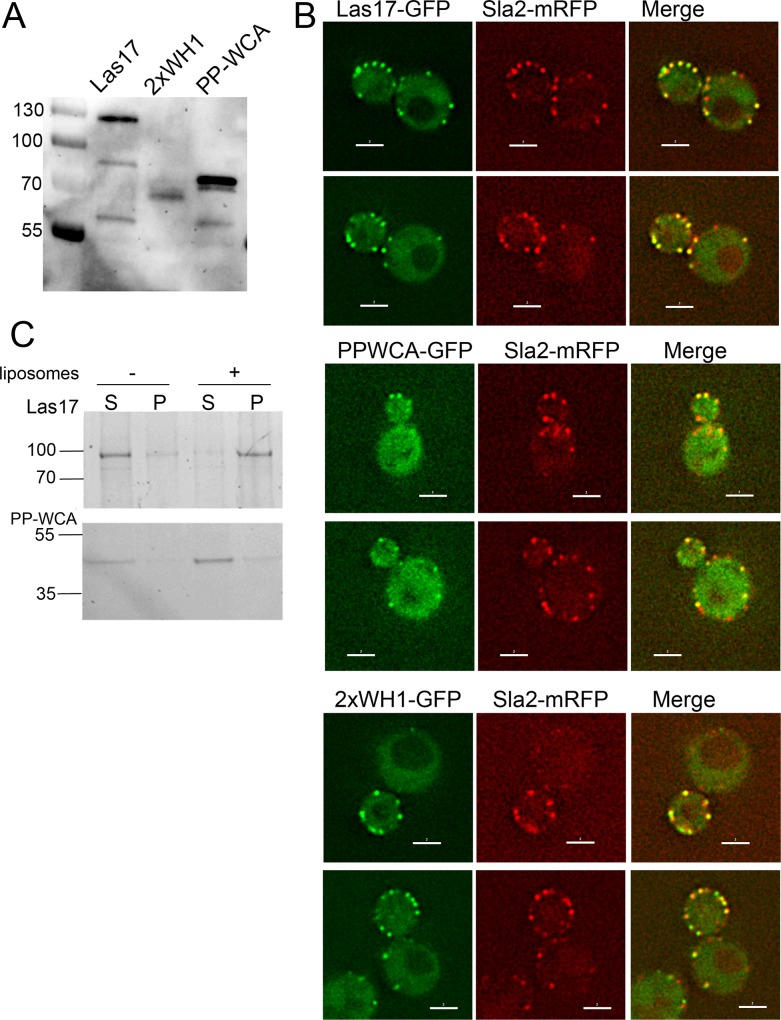
**N-terminal region of Las17 mediates localization to membranes** (A) Constructs expressing full length Las17, PP-WCA or 2xWH1 fused to GFP were expressed in yeast and extracts tested for expression using antibodies against GFP. (B) Localization of the GFP constructs were assessed in cells co-expressing the endocytic site marker Sla2-mRFP. Top panels: full length Las17-GFP, middle panels PP-WCA-GFP; lower panels, 2xWH1-GFP. Bar = 2 μm. (C) Full length Las17-myc purified from yeast and the recombinant PP-WCA Las17 fragment (300–633) purified from *E*.*coli* were incubated with liposomes before centrifuging to assess binding. S-supernatant and P–pellet fractions. Size of MW standard markers are marked in kilodaltons.

To address which regions of Las17 are critical for its localization we generated constructs carrying full length Las17, the PP-WCA fragment or the N-terminal WH1 fused to GFP. With the latter fragment distinct localization was not observed however, a construct expressing tandem WH1 domains showed a specific localization pattern and this construct was used for the comparison. Extracts expressing the constructs were checked for expression of the fusion (Fig 7A). Localization was then assessed in strains carrying Sla2-mRFP as a marker of endocytic sites. As shown in Fig 7B wild type full length Las17 shows a high level of co-localization with 85% sites containing both Sla2 and Las17. In contrast, the PP-WCA fragment shows a much higher cytosolic level of protein and a significantly reduced level of co-localization (45%). The N-terminal WH1-GFP gave a similar high level of co-localization as the full length protein (78%) indicating that this domain has the capacity to recognize endocytic sites even in the absence of its known SH3 domain binding site region. The high level of co-localization also suggests that recruitment is not solely mediated by the known WH1 binding partner verprolin (Vrp1) as this arrives much later than Sla2 and has a much shorter lifetime within a patch.

It is known that the WH1 region bears some homology to PH domains, and also that the interaction of PH domains with membranes can be enhanced if the domains are dimerised [16]. This suggests that the interaction of the WH1 domain with endocytic sites might be lipid mediated. To test binding of Las17 to membranes, full length Las17 was purified from yeast and incubated with pre-formed liposomes. A clear shift to the pellet fraction was seen in the presence of liposomes indicating binding. The PP-WCA portion of Las17 was also tested for its ability to bind to lipids but no binding was seen with the same liposomes supporting the idea that the lipid binding capacity of Las17 lies in its N-terminal region.

## Discussion

Mechanisms governing nucleation of actin filaments de novo are critical in order to allow filaments to form at appropriate sites in cells. While we have an in depth understanding of how Arp2/3 generates actin filaments from the sides of existing filaments [17–20], our knowledge of the source of the mother filaments required for Arp2/3 activity is still in its infancy. There is growing evidence for the role of tandem actin-binding domain proteins functioning in this regard, and while less effective at nucleation and driving rapid elongation of filaments, it seems likely that in many cases such proteins may be the source of filaments required to recruit and thereby support activation of Arp2/3 [5, 21].

WASP family proteins have been largely studied in a role as nucleation promotion factors with their C-terminal WCA domains being considered the primary functional part of the proteins, while other domains (e.g WH1, CRIB, polyproline) contribute to regulation or function as scaffolds for recruitment of SH3 domain proteins [4, 7, 22]. It was therefore surprising when deletion of the C-terminal domain of yeast Las17 had little discernible effect on actin polymerization required to support endocytosis [23, 24]. In this study we have demonstrated that WCA domain is able to contribute to the elongation of filaments generated through an Arp2/3-independent route, though this appears to be largely as a function of recruiting actin monomer and delivering to actin seeds generated by the poly-proline region. In the presence of Arp2/3, the contribution of WCA is significantly greater, though clearly both PP and WCA are functioning together and neither alone is as effective at promoting nucleation.

Feliciano and colleagues identified G-actin binding domains within the polyproline region and both we, and they, demonstrated the importance of these for Arp2/3-driven actin polymerization [11]. However, in the absence of Arp2/3 we have also shown that mutations affecting this G-actin binding have more severe phenotypes, with an RRRR(349,350,382,383)AAAA mutation significantly reducing actin nucleation activity in vitro. This data gives rise to the possibility that the paired basic residues within the polyproline region are in fact responsible for the nucleation involved in the initial de novo generation of filaments rather than simply serving to funnel monomers for Arp2/3-dependent functions. Our in vivo data analyzing behaviour of endocytic reporters suggests that this is the case, as striking defects are detected prior to recruitment of Arp2/3 (Fig 5). The binding analysis does however indicate that there is still clear actin binding possible in the presence of the paired arginine mutations (Fig 3A). This suggests that there is further actin interacting capacity within the fragment that awaits further analysis. An important consideration for future studies arising from our work is the interplay between the SH3 domains known to interact with the proline tracts with flanking arginines in Las17 [14], and the actin nucleating properties of the region. An exciting possibility is that SH3 domains are directly regulating the actin nucleating function of Las17 independently of Arp2/3.

The role of the paired basic residues in actin nucleation does however raise a question as to why previously the proline tracts themselves appeared important both in vitro and in vivo for effects on actin polymerization and function. Our data here suggests that the proline tracts function in tandem with the actin nucleating activity within the polyproline region to effectively stabilize or tether the F-actin nucleus and nascent filament. Mutation or absence of proline tracts reduces filament elongation presumably because nuclei are generated but are released to the medium or cytosol. This inability to effectively tether nascent filaments might also explain why the Las17 proline mutation (PP506,507AA) caused such a severe defect in vivo [8]. In this mutant, if the nascent filaments aren’t tethered or stabilized at the site of formation, then it is simply much more difficult to establish a site for subsequent invagination events.

A final essential aspect of Las17 function is how it is recruited to appropriate places for its function. Work in a recent paper demonstrated how the essential endocytic functions of both Las17 and the type-1 myosin proteins of yeast can be replaced by a single engineered fusion of the Myo5 and Las17 [25]. Critical domains in this fusion include the myosin motor and TH1 lipid-binding domains and the region 324–426 of Las17. This analysis therefore highlighted the requirement for nucleation function to be tethered to the membrane. Given that we can detect clear membrane binding by purified yeast Las17 we would consider that its own lipid-binding functions could alternatively support this requirement for membrane binding within the fusion. In addition, our analysis of WH1 domain localization suggested that GFP-localization to the plasma membrane was only detectable when using a construct with tandem WH1 domains. Given the fact that other lipid binding domains, such as those in dynamin, also show a need for dimerization in order to interact with membranes [16], the data might suggest that Las17 itself dimerizes, or that another protein binds to more than one Las17 to effectively bring tandem WH1 domains to the membrane for an interaction. Currently, these ideas are speculative but suggest that further investigation in this area could have significant impact on our understanding of Las17 and WASP family proteins.

Taken together we propose a model in which Las17 is recruited to endocytic sites, partly driven by an affinity for lipids. Once at the endocytic site, and possibly regulated by an interaction with lipids, the paired basic residues in the polyproline region are then responsible for binding actin monomers and initiating formation of a nucleus possibly in a similar manner to that proposed for WH2 domains in Spire [21, 26]. The concomitant binding of the F-actin nucleus and the nascent filament by C-terminal proline tracts ensures that new actin monomers can be added without release of the nucleus itself. Within the context of this Arp2/3-independent actin nucleation and elongation, the WH2 domain serves to bind monomer and effectively deliver it to the region of filament growth but this monomer itself is not part of an initial filament nucleus.

While this study focuses on Las17 the WASP ortholog in yeast, the role of the polyproline regions in the other WASP family proteins remains poorly characterized. In particular, significant questions remain as to the role played by the polyproline regions in generating actin filaments both in the presence and absence of Arp2/3. Furthermore, the growing recognition that internal cell membranes such as endosomes and autophagosomes require actin to facilitate protein sorting and membrane organization highlights a need to gain a greater understanding of the mechanisms underlying the formation of new actin filaments at membranes.

## Experimental Procedures

### Molecular biology and yeast methods

Plasmids used in this study are listed in Table 1. Generation of mutants was achieved using the QuikChange mutagenesis kit (Stratagene) according to manufacturer’s instructions. Yeast strain generation. Las17 sequence carrying the RR349,350AA, RR383,383AA mutations was introduced into the yeast genome by allele exchange. *Las17* (including flanking sequence 40 bp upstream and downstream) bearing the mutation was amplified by PCR, along with a selectable *URA3* marker. The PCR product was transformed into yeast strain with *las17* deleted with *URA* marker (KAY1801: KAY1888: Mat**a**, *ura3-52*, *leu2-3*,*112*, *his3*Δ*200*, *trp1-1*, *lys2-801*, *las17*::*URA3*) using a lithium acetate based protocol. Cells were plated onto selective plates lacking uracil and containing 5-FOA (5-Fluoroorotic Acid). Allele exchange was verified by colony PCR and the mutation confirmed by sequencing. Strain generated KAY1888: Mat**a**, *ura3-52*, *leu2-3*,*112*, *his3*Δ*200*, *trp1-1*, *lys2-801*, *las17RR349*,*350AA*, *RR382*,*383AA*.

**Table 1:** Plasmids used in this study.

Plasmid	Description	Source
pKA417	pGEX-6P1 (all GST fusions below were generated in this plasmid)	GE Healthcare
pKA1061	GST-Las17 180–633	This study
pKA566	GST-Las17 300–422	[27]
pKA1189	GST-Las17 300–422 RR349,350AA	This study
pKA1190	GST-Las17 300–422 RR382,383AA	This study
pKA1191	GST-Las17 300–422 RRRR349,350,382,383AAAA	This study
pKA670	GST-Las17 300–536	[8]
pKA958	GST-Las17 300–536 PP506,507AA	This study
pKA671	GST-Las17 300–633	[8]
pKA1045	GST-Las17 300–633 RR349,350AA	This study
pKA1173	GST-Las17 300–633 RR382,383AA	This study
pKA1174	GST-Las17 300–633 RRRR349,351,382,383AAAA	This study
pKA1186	GST-Las17 414–633	This study
pKA1080	GST-Las17 460–633	This study
pKA1079	GST-Las17 490–633	This study
pKA1214	GST-Las17 414–536	This study
pKA1013	GST-Las17 529–633	This study
pMW172	pET derived vector with untagged profilin cloned into polylinker for bacterial expression	[28, 29]
pAR55	pRS426 pGal LAS17 TEV 9xmyc	[10]
pKA607	pTPI1 LAS17-GFP, LEU, CEN	This study
pKA712	pTPI1-Las17(1–174+1–152)–GFP LEU, CEN	This study
pKA1239	pTPI1-Las17ppWCA–GFP(residues 300–633); LEU, CEN	This study

Sla1-GFP and Arc15-mCherry were tagged in the genome of wild-type cells and cells carrying Las17 RR RR mutation sequentially. PCR product containing the GFP or mCherry sequence flanked by approximately 50 base pairs of sequence homologous to the chromosomal site of insertion was introduced the cell by direct transformation. For isolation of successful transformants, an insert contained *TRP1* and *HIS3* selection marker respectively. In addition, insertion of a tag was confirmed by colony PCR and by fluorescence microscopy. Strains generated

KAY1878 (Mat a, *ura3-52*, *leu2-3*,*112*, *his3*Δ*200*, *trp1-1*, *lys2-801*, *Sla1-GFP*::*TRP1*, *Arc15-mCherry*::*HIS3*) and KAY1891 (Mat a, *ura3-52*, *leu2-3*,*112*, *his3*Δ*200*, *trp1-1*, *lys2-801*, *las17RR349*,*350AA RR382*,*383AA*, *Sla1-GFP*::*TRP1*, *Arc15-mCherry*::*HIS3*). To visualize Las17 fragment localization, plasmids pKA607, pKA712 and pKA1239 were transformed in KAY1400 *MATα*, *ura3-52*, *leu2-3*,*112*, *his3*Δ*200*, *trp1-1*, *lys2-801*, *Sla2-mRFP*::*HIS3*

Transformations into yeast were performed using lithium acetate as described previously [30]. Cells were grown with rotary shaking at 30°C in liquid YPD medium (1% yeast extract, 2% Bacto-peptone, 2% glucose supplemented with 40 μg/ml adenine) or in synthetic medium (0.67% yeast nitrogen base and 2% glucose), with appropriate supplements. Solid media was made by addition of 2% agar. Yeast whole cell extract**s** were prepared from 5.0 OD_600_ units of yeast in liquid medium and separated by SDS PAGE (Any kD™ Mini-PROTEAN^®^ TGX™Gel, BioRad). Untagged Las17 was detected on gels using affinity purified anti-Las17 antibodies raised to the PP-WCA fragment (CamLab, UK). Las17-GFP construct expression was analysed using monoclonal anti-GFP antibodies (Roche, clone 7.1and 13.1 mix) at 1:500 dilution: Halo assays were performed as previously described [12].

### Protein purification

Las17 fragments were expressed as GST fusion proteins as described previously [8]. Briefly, GST-expression plasmids were transformed into Lucigen OverExpress C41(DE3) cells. Protein was purified from 2 l of cells after induction by isopropyl β-D-1-thiogalactopyranoside (5 hr 37°C). Lysates were incubated with glutathione Sepharose beads (GE Healthcare) for 1 hr at 4°C, washed, and proteins were cleaved using PreScission Protease according to the manufacturer’s instructions. Cleaved protein was buffer exchanged into G-buffer (2 mM Tris-HCL [pH 8.0], 0.2 mM CaCl_2_, 1 mM NaN_3_, 0.5 mM dithiothreitol, 0.2 mM ATP), kept on ice at 4°C, and used within 24 hr. Peptides used, Las17(501–528) were synthesized by Pi Proteomics (Huntsville, AL) and are as previously described [8].

Full length Las17 was expressed in *S*. *cerevisiae* as a Myc fusion protein and purified using a modification of the method described [10]. 20 ml of overnight culture of pKA516 (Gal-Las17-Fl-9xMyc) in BJ1991 cells was used to innoculate 1L SD-Ura raffinose to 0.1OD_600_ and grown with agitation at 30°C for 8 hrs. The media was supplemented with 20 g bacto-peptone, 10 g yeast extract and 2% (v/v) galactose and growth was continued at 30°C overnight, before harvesting by centrifugation. The pellet was resuspended in 10 ml extraction buffer (100mM HEPES pH 7.5, 1mM EDTA, protease inhibitor cocktail, 1mM PMSF, and the cells broken by passing twice through a French press at 9000–10000 psi. 0.5% Triton x-100 was added and the extract left on ice for 10 mins before centrifugation at 21000 x g for 20 mins at 4°C. The supernatant was decanted into a fresh tube and the spin repeated. The supernatant was added to 400 μl anti-Myc agarose beads (25% slurry) (previously washed three times with PBS and equilibrated in extraction buffer + 0.5% Triton X-100), and rolled at 4°C for 1.5 hrs. Beads were harvested by low speed centrifugation and washed with 10ml 20mM HEPES (pH7.5), 1mM EDTA, 0.5% Triton X-100, then with 10ml 20mM HEPES (pH7.5), 1mM EDTA, 500mM KCl, and finally with 10ml 20mM HEPES (pH7.5), 1mM EDTA, 50mM KCl. Beads were resuspended in 200 μl of the final wash buffer and transferred to a microcentrifuge column. 7U TEV protease was added and incubated at room temperature with occasional agitation for 1.5 hrs. A further 7U TEV protease were added and beads were left for a further 1hr. Cleaved Fl-Las17 was analysed by SDS-PAGE

Rabbit skeletal muscle actin was purified and gel filtered as described [31].

Untagged yeast profilin was obtained in a plasmid pMW172 as a gift from Bruce Goode. It was expressed in BL21(DE3) cells and purified as described for non-fusion cofilin, except that the pH was adjusted to 8.0, and profilin was eluted from the Q-sepharose column with a stepped gradient of 50 mM increments from 0 to 1M NaCl over a total of 200 ml [32]. Fractions containing profilin were analysed by SDS PAGE, pooled, concentrated to 4 ml in an Amicon Centriprep (3kDa cut off), buffer exchanged into G-buffer (2mM Tris pH 8, 0.2mM CaCl2, 0.5mM NaN3) without ATP or DTT, and frozen in aliquots stored at -20°C.

Yeast actin was purified from wild type yeast obtained from Sainsbury’s supermarket. Cells were resuspended in yeast G-buffer (10mM Tris pH 7.5, 0.2mM CaCl2, 2mM DTT, 0.5mM ATP, 0.5mM NaN3) and broken by passing once through a cell disruptor (Constant systems) at 35 psi, frozen in liquid nitrogen and stored at -80°C until use. 100g of broken cells were thawed in 100ml of yeast G-buffer, spun at 185,000 x g for 30 mins, and the supernatant passed through a 0.2uM filter before loading onto a 30ml DNAse1 affinity column at 2ml^min-1^ which had been previously equilibrated in yeast G-buffer. The column was washed with 50ml yeast G-buffer, 50ml 5% formamide in yeast G-buffer, 5% formamide supplemented with 0.2M NH4Cl in yeast G-buffer, and finally 50ml yeast G-buffer only. Yeast actin was eluted with 50% formamide in yeast G-buffer and collected in 5ml fractions. Fractions containing protein were identified by spotting 10ul onto 3mm paper and staining with coomassie. These fractions were dialysed separately overnight against Yeast G buffer to remove the formamide. Fractions in which only actin was visible on a 15% SDS PAGE gel were pooled and concentrated to 1ml using a Centriprep concentrator (3kDa cut off), and aliquots were frozen in liquid nitrogen and stored at -80°C.

### Protein assays

For fluorometry assays, 370 μl assays used 3 μM actin as indicated unless otherwise specified (Fig 6D and 6E). Pyrene-actin was added to 5%, mixed thoroughly, and added to the fluorometry cuvette. Polymerization salts, 10× KME (500 mM KCL, 10 mM MgCl_2_, 10 mM EGTA, 100mM Tris-HCl [pH 8.0]) were mixed with fragments or G buffer to give final concentration of 0.5× KME before reading fluorescence. Polymerization was observed in a Cary Eclipse fluorometer (emission 364 nm, slit 10 nm round; excitation 385 nm, slit 20 nm) as described previously [8]. Unless otherwise stated Las17 fragments were used at 0.3 μM and Arp2/3 (obtained form Cytoskeleton Inc, USA) at 5 nM.

Microscale Thermophoresis (MST) exploits a change in mobility away from a heat source to detect intermolecular interactions. Rabbit muscle actin in G-buffer minus DTT was labelled using Monolith NT^TM^ Protein Labelling Kit RED-Malemide (NanoTemper Technologies, Munich, Germany). Las17 was prepared as a two fold serial dilution and an equal volume of labelled actin was added to each reaction (Final buffer conditions: G-buffer + 0.05% Tween). Each reaction was then loaded into Monolith NT.115 Premium Treated Capillaries (NanoTemper Technologies) and thermophoresis was measured in a Monolith NT.115 Microscale Thermophoresis device (NanoTemper Technologies). Thermophoresis was measured at 20% red LED power and 40% IR laser power at 22°C. Fnorm was calculated from the ratio of fluorescence before heating and after the new equilibrium had been reached. Fnorm was then plotted against Las17 concentration to give the Las17 dependent change in thermophoresis. This was described by the ‘Kd fit’ model on the provided software to give Kds for actin binding to Las17 fragments.

Liposome binding assays. Liposomes were prepared and binding assays carried out as described [33]. For preparation of liposomes 11 μl of a 25 mg/ml solution of Folch fraction 1 (Sigma) was supplemented with 7.5% PI4P (Avanti polar lipids) and dried under a nitrogen stream. They were then washed in chloroform, dried and further washed with diethyl ether to remove all traces of chloroform before being dried again. The dried lipid mixture was resuspended in 200ul of buffer B (20mM HEPES pH 7.2, 100mM KCl, 2mM MgCl2, 1mM DTT) at 60°C for 30 min with regular gentle agitation. Liposomes were then extruded 11 times through polycarbonate filters with 1.0 μm pores. For liposome binding assays 20 μM Las17 (pre-spun at 313,000 x g for 15 min) was mixed with 20 μl liposomes in buffer B, and incubated at room temperature for 15 min. Liposomes and bound protein was pelleted by centrifugation at 250,000 x g for 15 min, and samples were analysed by SDS-PAGE and Coomassie staining.

### Microscopy approaches

Fluorescence microscopy was performed on an Olympus IX-81 inverted microscope with 100x and 150x oil objectives and data deconvolved using Iterative Deconvolve 3D plugin in Fiji software. To visualize formation of actin filaments in vitro, a reaction mix of 3 μM rabbit muscle actin with 0.4 μM Alexa 488 labelled actin and 0.3 μM relevant fragment of Las17 was prepared. Polymerization was induced by addition of polymerization salts. 30 minutes after the reaction was started a 30 X dilution was performed in F-buffer plus 10% glycerol and filaments were visualized. Filaments were measured and counted using ImageJ. To visualize F-actin organization in vivo, cells were fixed and labelled with rhodamine-phalloidin as previously described [34]. For live-cell imaging, cells were visualized in early log phase. Time-lapse live cell imaging of GFP and mCherry tagged proteins was performed with 1 sec time-lapse. The life-time of moving fluorescence spots was measured and arbitrary profile of intensity values using ImageJ.
